# Revisiting an Analysis of Threats to Internal Validity in Multiple Baseline Designs

**DOI:** 10.1007/s40614-022-00351-0

**Published:** 2022-07-26

**Authors:** Timothy A. Slocum, P. Raymond Joslyn, Beverly Nichols, Sarah E. Pinkelman

**Affiliations:** Utah State University, 2865 Old Main Hill, Logan, UT 84322 USA

**Keywords:** Single-case design, Multiple baseline design, Concurrent, Nonconcurrent, Research methodology, Internal validity

## Abstract

In our previous article on threats to internal validity of multiple baseline design variations (Slocum et al., 2022), we argued that nonconcurrent multiple baseline designs (NCMB) are capable of rigorously demonstrating experimental control and should be considered equivalent to concurrent multiple baselines (CMB) in terms of internal validity. We were fortunate to receive five excellent commentaries on our article from experts in single-subject research design—four of whom endorsed the conclusion that NCMBs should be considered strong experimental designs capable of demonstrating experimental control. In the current article, we address the most salient points made in the five commentaries by further elaborating and clarifying the logic described in our original article. We address arguments related to classic threats including maturation, testing and session experience, and coincidental events (history). We rebut the notion that although NCMBs are strong, CMBs provide an increment of additional control and discuss the application of probability-based analysis of the likelihood of threats to internal validity. We conclude by emphasizing our agreement with many of the commentaries that selection of single-case experimental designs should be based on the myriad subtleties of research priorities and contextual factors rather than on a decontextualized hierarchy of designs.

We received five thoughtful commentaries on our analysis of threats to internal validity in multiple baseline variations (hereafter referred to as the target article; Slocum et al., 2022), and are grateful to our colleagues who took the time and effort to respond to our analysis and extend the discussion to encompass new ideas and directions. In the target article, we made a case that, in spite of the common belief to the contrary, the nonconcurrent multiple baseline (NCMB) is as rigorous an experimental design as the concurrent multiple baseline (CMB). Ledford (this issue) and Smith et al. (this issue) agreed with our argument that there is no general hierarchy between CMBs and NCMBs. In fact, Ledford had arrived at similar conclusions independently of our article (see Ledford & Zimmerman, 2022). Horner et al. (this issue) and Kratochwill et al. (this issue) agreed that NCMBs should be considered fully rigorous research designs, but in general not as strong as CMBs. Kennedy (this issue), however, asserted that NCMBs do not control for the threats of coincidental events and instrumentation, and therefore cannot be considered fully rigorous designs on par with CMBs.

In this rejoinder, we address the most salient points made in the commentaries. First, we discuss comments on our interpretation of the specific threats to internal validity: maturation, testing and session experience, coincidental events, and instrumentation as they relate to CMBs and NCMBs. Second, we consider the value of the “across-tier” comparison, including its strengths and limitations. Third, we address the probability-based analysis of threats. Finally, we will discuss aspects of social validity, nuances of selecting experimental designs, and future directions for discourse on this topic.

## The Threat of Maturation

Kennedy suggests that maturation threats are rarely relevant to multiple baseline designs: “Given that most maturational variables unfold over the developmental course of the individual, they tend to be gradual (cf. Rosales-Ruiz & Baer, 1997). Therefore, as long as the multiple baseline design is relatively brief in time scope (e.g., days or weeks), developmental threats to experimental control may not be a concern.” This depends on how narrowly or broadly one defines the maturation threat. To be sure, much maturation is very gradual and could be negligible on the timescale of most SCR. In our article, however, we intentionally defined all the threats broadly. This is to encompass as many specific extraneous variables as possible within each type of threat. The goal is to address all plausible extraneous variables within the analysis of threats to internal validity. It would be a serious flaw if any plausible extraneous variable was not recognized in some category of threats and therefore were not addressed by the experimental design. We defined maturation as:

. . . extraneous variables such as physical growth, physiological changes, typical interactions with social and physical environments, academic instruction, and behavior management procedures that tend to cause changes in behavior over time . . . *the key characteristic that maturational processes share is that they may produce behavioral changes that would be expected to accumulate as a function of elapsed time in the absence of participation in research*. (Slocum et al., 2022, emphasis added)

**Fig. 1:**
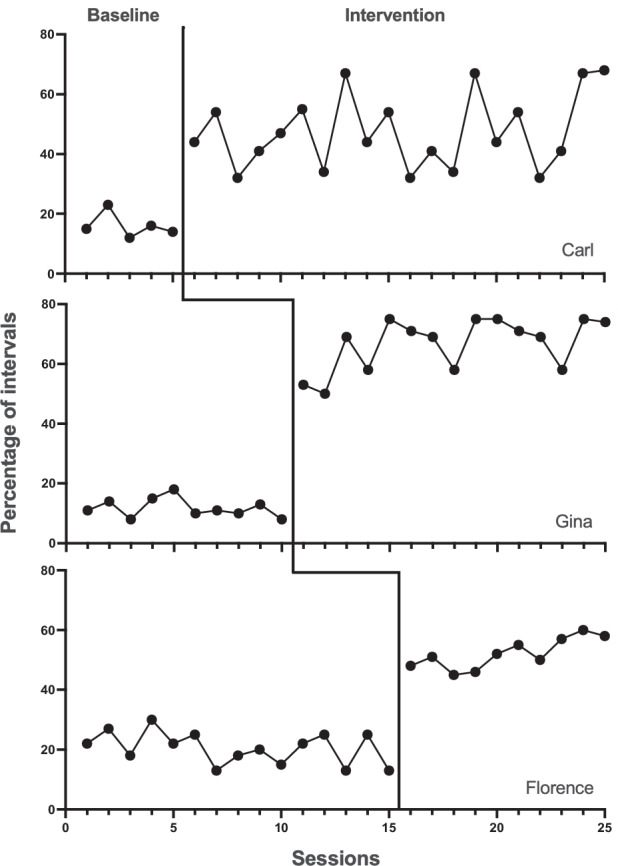
A Hypothetical Data Set Using a Multiple Baseline across Participants Design

It is critically important that we consider this broad class of extraneous variables as potential threats to internal validity of multiple baseline designs whether or not all instances meet an intuitive definition of maturation. Fortunately, controlling this broad class of potential threats is relatively straightforward: the researcher ensures that the tiers include substantially different amounts of time in baseline. Thus, no single length of time for maturation can account for the pattern of results expected in a multiple baseline design (see Fig. 1). This logic applies equally to CMB and NCMB designs. Both types of MB afford replicated within-tier comparisons and across-tier comparisons when addressing maturation.

## The Threat of Testing and Session Experience

Kennedy states that “test–retest” threats “are largely controlled for through the repeated administration of baseline sessions” (p. ##). Again, this is a narrower interpretation of the threat than we discussed and it is important to define threats as broadly as possible to increase the range of extraneous variables that we consider in our experimental design. We defined the threat of testing and session experience as “features of experimental sessions (both baseline and intervention phases) other than the independent variable that could cause changes in behavior” (p. ##). Given this more inclusive conceptualization of the threat, a stable baseline is not enough. In order to ensure that nonlinear changes associated with participation in baseline assessment and other aspects of sessions could not account for observed results, the tiers of a multiple baseline must include substantially differing number of baseline sessions. With this control in place, the effects of any combination of testing and session experience could not explain apparent treatment effects in three (or more) tiers of a multiple baseline (see Fig. 1). And again, this control is equivalent in CMB and NCMB; both offer within-tier and across-tier comparisons with respect to testing and session experience.

## The Threat of Coincidental Events

Kennedy states that history effects (i.e., coincidental events):

are simply not controlled for using a nonconcurrent multiple-baseline design. The concurrent multiple baseline design provides an effective experimental foil for such events because of the temporal synchronization of the design. However, I do not believe that nonconcurrent multiple-baseline designs do control for many possible history threats to experimental control although some instances can be (as noted by Slocum et al.). (p. ##)

**Fig. 2:**
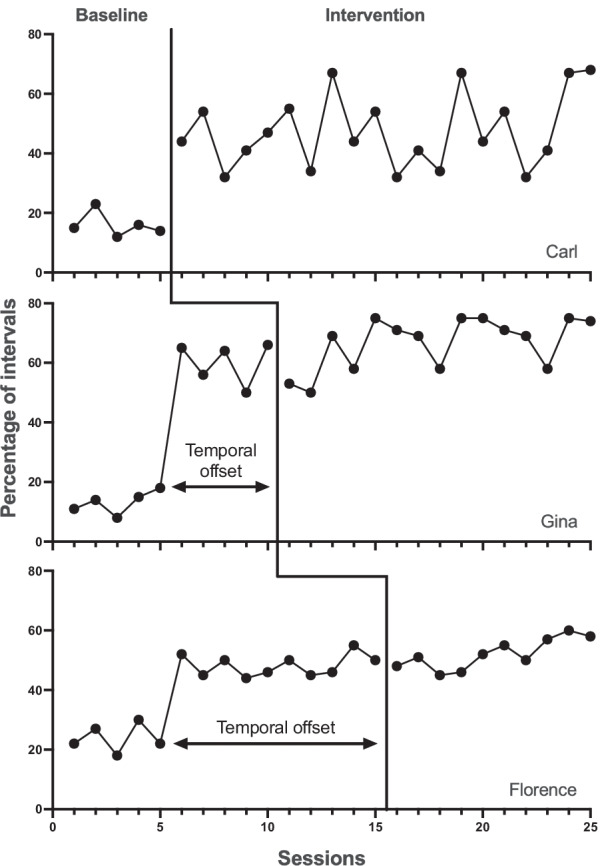
A Hypothetical Data Set Showing a Multiple Baseline across Participants Design Confounded by a Coincidental Event. *Note.* The temporal offset provided by the multiple baseline design is highlighted in tiers 2 and 3

Although his conclusion is clear and unambiguous, Kennedy gives no support for it. He points to no flaws in our reasoning and offers no logic of his own. Our logic is that given sufficient temporal offset of phase changes across tiers, no single coincidental event can produce a treatment-like change in more than one tier. Thus, it would require three distinct and perfectly timed coincidental events to account for the overall pattern in a three-tier MB (see Fig. 2). By the standard of the SCR community, this is not considered to be plausible (Kennedy, 2005). This logic is the same for both concurrent and nonconcurrent designs.

## The Threat of Instrumentation

Kennedy raises the issue of the threat of instrumentation, one that we did not address in our target article. We agree that this is a critical threat and are grateful that he raised it. Kennedy points out that in single-case research one important component of the treat of instrumentation is observer drift—that apparent treatment effects could be a result of human observers changing the way they code their observations of the dependent variable or recording system malfunctions. Kennedy states:

A time-locked [i.e., concurrent] multiple-baseline design controls well for instrumentation changes, but it is not clear that the nonconcurrent variant could accomplish a similar level of control. An example might be observer drift that occurs in only one-tier of the nonconcurrent multiple baseline design and not in others. In such an instance, there may be a disruption to experimental control in only one-tier of the design and not others, thus influencing the degree of internal validity of the experiment. (p. ##)

In his example, Kennedy posits observer drift that contacts only one tier of a multiple baseline. If that were the case, instrumentation could not explain changes in the other tiers, and thus would not threaten the overall conclusion. An event only threatens internal validity if it could account for changes in at least three tiers—that is, the overall pattern of results (e.g., see Fig. 1).

Still, it may be possible for observer drift to contact all tiers and threaten internal validity in at least three ways. One possibility is that observers are trained and reach the apogee of their accuracy immediately before baseline begins. Then, across time, they become less accurate and reliable. This would be controlled by varying the number of days in baseline. A second possibility is that observer drift occurs as a result of the experience of making observations in sessions. This would be controlled by arranging tiers with differing number of sessions in baseline. A third theory could be that observer drift occurs on a given day, perhaps as a result of some outside event. This would be controlled by ensuring that phase changes are substantially offset in calendar dates. The threat of instrumentation (observer drift) provides an excellent example of how the three dimensions of offsetting phase changes provide control for threats beyond maturation, testing and session experience, and coincidental events. Varying the number of days in baseline controls *any* threat that is a function of time elapsed in baseline, whether the particular threat involves changes in the participant, observers, aspects of the sessions, or variables that we have not anticipated. Varying the number of sessions in baseline controls *any* threat that is associated with sessions, again, without respect to what components of the experiment cause the change in the dependent variable. Likewise, manipulating the independent variable on substantially different dates across tiers controls for *any* extraneous variable that has (or begins to have) its effect on a single day. These experimental design features offer control for broad classes of threats including potential threats that the researchers (or readers) have not even considered.

## Does the Across-Tier Comparison Offer Additional Control?

Two responses to our target article (Horner & Machalicek, this issue; Kratochwill et al., this issue) agree with the logic of control for threats to internal validity in CMB and NCMB designs that we described in the original article. They agree that NCMB should be considered fully legitimate experimental designs capable of rigorously demonstrating experimental control and causal relations between independent and dependent variables. Nonetheless, these authors find CMBs to be incrementally stronger because they afford an across-tier comparison that is relevant to detecting coincidental events. These authors appear to suggest that if a researcher can obtain some modicum of additional information about coincidental events from the across-tier analysis, and this comes with no costs or disadvantages, it would be wise to take advantage of that incremental improvement.

If the extra information available in a CMB did, in fact, come without costs, we would agree. However, there are at least three kinds of potential costs to using a CMB based on the information provided by the across-tier comparisons. First, using the across-tier comparison may produce overconfidence in internal validity of the study. As we discussed in the original article, (1) the across-tier comparison is certain to fail to show the effects of tier-specific coincidental events because they contact only one tier (e.g., a person-specific event such as an illness would only contact one tier of a multiple baseline across participants); (2) the across-tier comparison may fail to show the effects of many other coincidental events that contact all tiers but have a distinct effect on only one tier (e.g., in a multiple baseline across settings, teasing on a bus ride to school could have a large effect on performance in a setting that is measured in the morning but no effect on settings measured later in the day); and (3) the across-tier comparison is considered meaningful support for internal validity when no change is seen in untreated baselines, but there is no way to determine if this absence of effect indicates that no coincidental event occurred or if there was a coincidental event that did not contact and have distinct effects on all tiers. As Ledford (this issue) noted, “the lack of evidence for threats to internal validity is weak evidence that they do not exist” (p. ##).

Second, the use of a NCMB can increase the temporal separation between phase changes in the successive tiers without having to hold any participant in baseline for an inordinately long period of time. This point was noted by Ledford (this issue). This longer temporal lag between phase changes would increase confidence that no single coincidental event could account for a change in more than one tier. Compared to a CMB with only 1–3 days of lag between phase changes, a NCMB with weeks or months of lag might confer significant improvements in control of coincidental events.

Third, there is a tricky dilemma in arranging a study to take advantage of the across-tier comparison. The across-tier comparison is most likely to reveal coincidental events (and less likely to be misleading) to the extent that all tiers contact the same potential coincidental events. But if the researcher designs tiers such that they are exposed to the same potential coincidental events, *they increase the chance of precisely the situation that is most dangerous to internal validity—a coincidental event operating on all tiers*. The only thing that stands between this situation and a complete failure of internal validity is the temporal lag between phase changes. The temporal offset alone ensures that the event that coincides with a phase change in one tier occurs during baseline in a later tier and is detected. So, the researcher must have a great deal of confidence that the temporal lag will allow them to distinguish the effect of the coincidental event from that of the IV. But if the offset is sufficient to make this distinction, then the within-tier comparison (available in a NCMB) will provide sufficient control (i.e., no single coincidental event could account for an apparent treatment effect in more than one tier) and one does not need the across-tier analysis. That is, if the researcher is not confident in the temporal offset of tiers exposing coincidental events, they cannot trust the vertical analysis; but if they are confident in the temporal offset, the replicated within-tier analysis is conclusive.

In addition, researchers and consumers of research should be aware of the specific design features required for the across-tier comparison to be valid. In a CMB, all tiers must be synchronized throughout the duration of the study (or at least the period of time during which one is concerned about the effects of a coincidental event). It is not sufficient that all tiers begin baseline on the same day. In our original article, we specified, that sessions must be synchronized across tiers such that Session 1 must take place in all tiers before Session 2 could take place in any tier, and this pattern (i.e., all tiers experiencing Session X before any tier experiences Session X+1) must be maintained throughout the study. When multiple sessions are conducted on a single day, they must be coordinated in time. And when a session cannot be conducted in any tier (e.g., a participant is absent in an across-participant design), the researcher must consider whether conducting sessions in other tiers will violate synchronization. This is a demanding standard, but strictly speaking, the across-tier (vertical) analysis is only valid when sessions are synchronized in this way. Otherwise, sessions that appear to be simultaneous could, in fact, be offset by several days. To be fair, it may be reasonable to exercise judgement about when synchronization is strictly necessary. It may not cause a practical problem to have minor violations that are far from the time at which the IV is manipulated. But strict synchronization is critical immediately before the phase change in each tier and in the offset period in which one tier is in treatment while another tier is still in baseline. Without this precise synchronization, the across-tier comparison could be flawed in an additional way. A single coincidental event could affect multiple tiers but these effects may not be aligned vertically on the graphs. With current reporting practices, readers can rarely be certain that this strict synchronization has been respected. Thus, the synchronization required of a CMB may be onerous for the researcher and if it is not strictly observed, a critical assumption of the across-tier analysis—that vertically aligned data points represent a single point in time—is not fulfilled.

Horner and Machalicek (this issue) offer an example scenario in which a CMB is able to detect a set of coincidental events that would not be detected by a NCMB. In this scenario, a study is evaluating the effect of a social skills program in a three-tier NCMB across participants who are in separate classrooms. The researchers implement the treatment in Classroom A in October, in Classroom B in November, and in Classroom C in January. Unbeknownst to the researcher, the three teachers share instructional materials including a single copy of a new social studies program that is engaging and enjoyable for the students. Because there is only one copy of the program, only one teacher can use it at time. As it happens, the teacher in Classroom A uses it first and coincidentally begins using the program at the same time that the experimental treatment is implemented. Then, the teacher is Classroom B gets their turn to use the program and they begin using it coincident with the experimental phase change in their classroom. Finally, the academic program is passed to the teacher of Classroom C and they start teaching it on the same day that the experimenter implements the treatment in that classroom. As a result, all three classrooms show an apparent treatment effect that actually resulted from the instructional materials shared by the teachers. However, the proposition that each of the three teachers began using the instructional program at precisely the same time that the treatment was implemented in their tier is implausible. This scenario depends upon three coincidences, which is beyond community standards for what experimental designs must withstand (e.g., Horner et al., 2005). No three-tier multiple baseline design (nor ABAB design for that matter) can withstand three coincidences. In this scenario, the NCMB fails, not because it is nonconcurrent, but because of the timing of the three coincidental events. Suppose the researchers had sufficient resources and patience to collect data in all tiers continually from October through February, converting the design to a CMB with an extremely long lag between phase changes, but allowing for a proper across-tier analysis. Given the timing of the coincidental events, the CMB would fail in exactly the same way as the NCMB. The perfect timing of teacher program use could also confound a CMB with shorter, more traditional lags if the teachers coincidentally initiated use of the program at the time of each phase change. The problem illustrated in this scenario is not based on nonconcurrence; it only shows that three perfectly timed coincidences will break any design that is based on demonstrating three apparent treatment effects at three different points in time.

We are *not* arguing that NCMB are stronger or should be preferred; simply that CMBs should not be favored in a general sense. The choice among designs should be made based on the specifics of a particular study. On this point we concur with many of commentators (Horner & Machelicek, this issue; Kratochwill, et al., this issue; Ledford, this issue; Smith et al., this issue) who also emphasized that the choice of experimental design is nuanced and should be based on the specific parameters of a study.

## Probability-Based Analysis of Number of Tiers and Control of Threats

Smith et al. (this issue) describe Christ’s (2007) probability-based analysis of how the number of tiers and the number of sessions in each tier affect the probability of coincidental events correlating with the phase changes in each tier. This analysis demonstrates that, if (a) coincidental events occur randomly in time, and (b) no single coincidental event can cause a change in more than one tier, then the probability of coincidental events correlating in time with phase changes in a multiple baseline design becomes exceedingly small with a reasonable number of tiers and data points. (We recommend reading Smith et al. and Christ (2007) for the full analysis.) This is an important analysis that formalizes and quantifies the basic logic of replicated within-tier comparison with respect to coincidental events. However, we should be careful about the assumption of random distribution of coincidental events—even coincidental events can be nonrandom in time. For example, in many contexts the days of a week are not equivalent—Mondays may be affected by the preceding weekend, certain events during a workweek may occur on specific days, and Fridays may be affected by anticipation of the upcoming weekend. There may also be events associated with monthly cycles, school terms, and so on. Thus, we consider this analysis to be extremely helpful in appreciating the general magnitude of probabilities of coincidental events accounting for multiple baseline results, but we would not interpret them as *precise* measures of the probabilities of coincidental events accounting for observed results in a particular study.

Smith et al. extend Christ’s argument to the question of whether tiers must have a substantially different number of baseline data points (one of our defining characteristics of multiple baseline designs). They state:

Researchers could conceivably increase the number of tiers, the number of data points in each tier, or both to produce such low probabilities of extraneous variables causing each change in the DV that researchers may not need to stagger the number of *sessions* prior to IV implementation across tiers. The argument based on simple probabilities suggested by Christ (2007) does not rely on the assumption that there are a *different* number of sessions prior to IV implementation in each tier. (pg. ##)

Above, we were careful to discuss Christ’s analysis as it applies to the threat of coincidental events and not extend it to other threats that may systematically violate the assumption that extraneous variables have their effects at random points in time. If an extraneous variable affects a DV as a function of time spent in baseline (maturation and other possible time-based variables) or sessions spent in baseline (testing and session experience as well as other session-based factors), these effects are not random in time. That is, they may occur systematically after a certain amount of time or a certain number of sessions. If the extraneous variables in question are not randomly distributed, Christ’s analysis would not apply. For example, if a participant in a study of sight word reading engages in multiple sessions of testing in which they are asked to read words (even without feedback), this might affect their reading or responsiveness to later instruction. (In fact, the first author has seen this happen.) Smith et al. anticipate this issue and state:

A primary counterargument may be that, as pointed out by Slocum et al. (2022), the lag in IV implementation is the primary way for ruling out confounds related to maturation and testing. However, as we noted previously, it is never possible to rule out all potential confounds, so an experimental design should only need to rule out confounds that are likely to affect a given experiment based on the nature of the IV, DV, or both, to convince someone of an experimental effect.

Experimental designs are strong to the degree that they rule out large and important *categories* of possible extraneous variables (threats to internal validity). Researchers must consider the likelihood of threats to each particular study and optimize their design to address the most likely and most severe threats, but we must also recognize that our ability to anticipate relevant threats and estimate their likelihoods is limited. Therefore, stronger designs rule out threats that might be considered only marginally likely and those which have not been anticipated at all. This is why we suggested that multiple baseline designs be defined as “a single-case experimental design that evaluates causal relations through the use of multiple baseline-treatment comparisons with phase changes that are offset in (1) real time (e.g., calendar date), (2) number of days in baseline, and (3) number of sessions in baseline.” (p. xx) These three features *categorically* address variables that occur (1) randomly in time, (2) as a function of days in baseline, and (3) as a function of sessions in baseline. As we indicated above, these controls extend beyond particular threats that are frequently discussed to any other known or unknown extraneous variables that have these characteristics. That is, designs that meet this definition require fewer assumptions about extraneous variables than designs that do not meet it.

Nonetheless, we do recognize that no design is perfect. “Ideal” designs are not applicable to every important research context and it is important for researchers to have flexibility to conduct the best study that is possible in their context. Sometimes, in order to conduct the best possible study in a particular context, a researcher must make additional assumptions that given classes of extraneous variable are not plausible threats. Although there may be a strong argument that these assumptions are well-justified, this is not as strong as using a design that obviates the need for these assumptions. Therefore, we recommend that a clear distinction be made between designs that meet our definition of multiple baseline, and those that lack the required offset in baseline days, baseline sessions, or both. These designs could be referred to as *quasi-multiple baseline designs*^1^*—single-case experimental designs that evaluate causal relations through the use of multiple baseline*–*treatment comparisons with phase changes that are offset in real time (e.g., calendar date)*. For a quasi-multiple baseline design to be convincing, the researcher must make an explicit case that the fact that baselines have equal number of sessions and/or extend for equal amounts of time do not allow for plausible threats to internal validity.

## Social Validity outside the Single-Case Research Community

Smith et al. refer to issues of single-case research being understood and valued outside of behavior analysis. We believe that it is extremely important to recognize that the single-case research community is larger than behavior analysis and growing. For example, important primary studies, systematic reviews, and methodological papers are published in journals that are not specific to behavior analysis and may have readerships with little crossover with the *Journal of Applied Behavior Analysis*. For this community, the issues of understanding experimental control in MB designs is the same as it is within behavior analysis—we need to have more discussions such as the present one. The acceptance of single-case research outside of this broader single-case research community is a distinct issue. For this, we suggest a three-part approach. First, single-case researchers should continue robust discussions that promote continual critical reflection on traditional understandings of our methods and engagement with issues of importance to the broader social science research community. Several recent examples include the exchange on replication failures (Hantula, 2019), statistical analysis (Jarmolowicz et al., 2021), and factors related to the reliability and validity of visual analysis (e.g., Ninci et al., 2015; Wolfe et al., 2016). Second, the consensus of single-case researchers needs to be summarized in standards (written to include the kind of flexibility suggested by Smith et al. and Ledford). We believe that the What Works Clearinghouse (WWC) standards may have taken on an outsized importance in evaluating the rigor of single-case research because active single-case researchers have not achieved consensus on an alternative. If such an alternative existed, researchers could point to them in discussing the rigor of a particular study or set of studies with colleagues outside of the research tradition as well as with those inside it. Because such standards do not exist, WWC standards have, at times, filled the void and become the default for some journal reviews, grant applications, and systematic reviews. The WWC standards are not promulgated as universal standards for high-quality research, but rather as a statement of what is necessary to be included in the reviews that WWC conducts. WWC reviews are committed to combining quantitative effect sizes from single-case and group research, and doing so requires single-case studies to have certain characteristics that may not be required for other purposes. But as long as no other standards achieve an equally high level of social validity, application of the WWC standards is likely to be overgeneralized. Further, recent iterations of WWC standards (e.g., version 4.1) have been criticized by researchers and methodologists who have authored previous versions (Kratochwill et al., 2021) and conducted numerous systematic reviews (Maggin et al., 2021). The issues raised in these papers cast doubt on the wisdom of following these standards in order to increase the social acceptance of single-case research. These standards should not be confused with a statement of what constitutes high quality single-case research.

## How Should Researchers Select a Design?

We want to make it absolutely clear that we completely agree with Horner et al.’s (this issue’s) statement, “Humility about the large array of potentially confounding variables should lead us to opt for the most rigorous design option practicable.” We are *not* arguing for a reduction of rigor; we *are* arguing that NCMB can be every bit as rigorous as CMB. We are *not* arguing that we can anticipate all extraneous variables that can threaten the internal validity of a MB study; we *are* arguing that the three critical features of a MB (along with other features that are common to other single-case designs) are capable of controlling many plausible threats, anticipated and unanticipated.

We agree with the numerous comments across all the response papers that researchers should select (or construct) designs based on the nature of their questions and the many contextual variables specific to their study. We welcome Smith et al.’s (this issue) reminder that internal validity is only one of many values to be optimized in research design. Depending on their questions, researchers may emphasize external validity or ecological validity at the expense of some amount of internal validity; they may emphasize control for certain threats that are particularly likely over those that appear to be less likely; or they may be at a point in the development of a program of research at which emphasis on tight control of internal validity is not yet possible or not necessary. We also welcome Ledford’s (this issue) and Smith et al.’s (this issue) excellent point that the methodological strength of a study should be evaluated within the context of the specifics of that particular study, not against inflexible standards that fail to recognize context. The overall conclusion of our previous article is exactly this—the choice between CMB and NCMB should be based on research questions and situational specifics, not an abstract notion that one of these designs is inherently more rigorous. It is exactly the latter notion that would tend to distract researchers from humility, flexibility, and specificity in the face of the challenges of applied research. We find no compelling case in any of the response papers that would support opting for CMB as a generally preferred alternative.

## Future Directions for Discourse on Single-Case Research Methodology

There are many more topics of methodology for multiple baseline designs that need to be explored, debated, and discussed more thoroughly. As mentioned previously, current reporting practices often make it difficult to determine important characteristics of design methodology and Ledford (this issue) suggests that specific reporting guidelines be developed for multiple baseline designs. We strongly agree and look forward to discussion of both textual reporting and development of graphic conventions that better represent the three critical dimensions of phase change offset in multiple baselines. In addition, questions of predetermining baseline length (Ledford, this issue) and randomization of the timing of intervention (Kratochwill et al., this issue) are important issues that deserve extensive focused discussion based on specific methodological advantages and disadvantages. We hope that this special section will advance the understanding of internal validity issues of CMB and NCMB and perhaps encourage similar exchanges on other issues of SCR methodology.

## Footnotes

1 We suggest the term “quasi-” because this is parallel to the relation between group experimental designs (randomized control trials) and quasi-experimental designs. The latter lack the categorical control over a range of known and unknown extraneous variables conferred by random assignment, and instead the researchers must make a case that their procedures and observed similarity of groups at pretest minimize plausible threats to internal validity.
